# Ideophones in Japanese modulate the P2 and late positive complex responses

**DOI:** 10.3389/fpsyg.2015.00933

**Published:** 2015-07-02

**Authors:** Gwilym Lockwood, Jyrki Tuomainen

**Affiliations:** ^1^Department of Neurobiology of Language, Max Planck Institute for Psycholinguistics, NijmegenNetherlands; ^2^Division of Psychology and Language Sciences, University College LondonUK

**Keywords:** sound-symbolism, Japanese, ideophone, event-related potential, P2, cross-modal correspondence, synaesthesia/synesthesia

## Abstract

Sound-symbolism, or the direct link between sound and meaning, is typologically and behaviorally attested across languages. However, neuroimaging research has mostly focused on artificial non-words or individual segments, which do not represent sound-symbolism in natural language. We used EEG to compare Japanese ideophones, which are phonologically distinctive sound-symbolic lexical words, and arbitrary adverbs during a sentence reading task. Ideophones elicit a larger visual P2 response than arbitrary adverbs, as well as a sustained late positive complex. Our results and previous literature suggest that the larger P2 may indicate the integration of sound and sensory information by association in response to the distinctive phonology of ideophones. The late positive complex may reflect the facilitated lexical retrieval of arbitrary words in comparison to ideophones. This account provides new evidence that ideophones exhibit similar cross-modal correspondences to those which have been proposed for non-words and individual sounds.

## Introduction

Sound-symbolism, most simply defined as “the direct linkage between sound and meaning” (Hinton et al., 2006, p. 1), has traditionally played a peripheral role in linguistics. The assumption of an iconic link between form and meaning conflicts profoundly with the principle of arbitrariness (de Saussure, 1959), which holds language as “a wholly symbolic system, [whereby] the elements of which are manipulated on an abstract level of representation” (Perniss et al., 2010, p. 1). The progression of formal linguistics is a fair indication that its foundations of arbitrariness are solid, but this is not to say that iconic and arbitrary systems in language cannot exist side by side. Wider recognition of the existence and extent of such words has been hindered until relatively recently by two main factors; a historically Eurocentric linguistic perspective, which has contributed to the assumption that the relative paucity of sound-symbolism in most Indo-European languages is reflective of human language as a whole (Perniss et al., 2010), and a disunity of description and definition (Dingemanse, 2012). However, the field has a rather more global perspective than it used to, and has settled on *ideophone* as the preferred term for lexical classes of words which exhibit non-arbitrary relations between their form and their meaning. Ideophones are more specifically defined as “marked words that depict sensory imagery” (Dingemanse, 2012), and are found in various natural languages (Firth, 1964; Diﬄoth, 1972; Childs, 1994; Hamano, 1998; Perniss et al., 2010; Dingemanse, 2012).

The study of sound-symbolism is enjoying a renaissance in psychology too. The best known example of purely sensory sound-symbolism is Ramachandran and Hubbard’s (2001) *kiki/bouba* experiment, based on Köhler’s (1947) observations using *maluma/takete*. Participants overwhelmingly linked the novel word *kiki* with a spiky shape and *bouba* with a round shape, regardless of their native language. Ramachandran and Hubbard (2001) argued that the connections which humans make between voiceless consonants and sharp contours, and between voiced consonants and round contours [as well as between vowels and object size (Johnson, 1967; Ohala, 1994)], are synaesthetic. Many similar behavioral studies followed, which focused on eliciting participants’ impressions of nonsense words (Ramachandran and Hubbard, 2001; Maurer et al., 2006), asking participants to associate tastes to vowel sounds (Simner et al., 2010), or investigating the names of cancer drugs and patients’ perceptions of their effectiveness (Abel and Glinert, 2008). Such behavioral studies are certainly interesting, and have shown that form to meaning mappings cover various different sensory systems and are relatively consistent across languages, which means that speakers of one language to apply the same correspondences to other languages with some degree of success (Revill et al., 2014). However, apart from a few studies on the perception of ideophones by naïve speakers (Berlin, 1994; Oda, 2000; Iwasaki et al., 2007b), the extensive ideophone literature in linguistics has mostly been overlooked. Very few studies use naturally occurring sound-symbolic words, and instead rely on nonsense words which have been deliberately constructed to maximize perceptual differences between conditions. While informative about the nature of synaesthetic effects between sounds and various sensory systems, this does not reflect sound-symbolism as it is used in real language.

Research in Japanese linguistics has proven to be more fruitful for examining sound-symbolism using natural language. Japanese has an extensive set of ideophones (known as *mimetics* in the Japanese literature; Kita, 1997; Hamano, 1998; Akita, 2009), which lend themselves to behavioral, developmental, and neuroimaging experiments where more specific hypothesis can be tested. Several studies have shown that naïve participants are sensitive to the association between ideophone form and ideophone meaning at above chance level, and that articulation facilitates this connection between form and meaning (Kunihira, 1971; Oda, 2000; Iwasaki et al., 2007b). However, Japanese sound-symbolism is not entirely transparent to other speakers. Iwasaki et al. (2007b) found that English speakers with no knowledge of Japanese could make accurate judgments about ideophones expressing certain semantic dimensions but not others (a finding which has been supported by corpus studies, such as Gasser et al. (2010), which have shown a correlation between phonetic form and semantic domain in ideophones). This suggests that while there is some solid evidence for cross-linguistic sound-symbolism, ideophones are not completely intuitive to speakers of other languages. Developmental literature suggests that this general cross-linguistic insensitivity to ideophones may be conditioned in adults by language’s arbitrary and conventionalized connections. Children are more sensitive to sound-symbolic patterns in Japanese than to arbitrary forms, and this sensitivity appears to function as a scaffold for language acquisition (Ishiguro, 1993; Iwasaki et al., 2007b; Imai et al., 2008). This finding is consistent across both Japanese and English-speaking children (Kantartzis et al., 2011; Yoshida, 2012), providing evidence toward a cross-linguistic – or, perhaps more accurately, language-independent – early sensitivity toward sound-symbolism.

EEG studies on sound-symbolism have found a variety of different effects at a variety of different time points, and so it is still unclear what the general effects of sound-symbolism are. Most EEG studies have used matching tasks, where participants are presented with images and sound-symbolic non-words which are either sound-symbolically congruent (e.g., *kiki* and a spiky shape) or incongruent. Kovic et al. (2010) found that congruent conditions elicited a greater negative-going wave at 140–180 ms, and that this effect was most prominent at occipital electrodes. Asano et al. (2015) tested preverbal 11-months-old infants, and found that incongruent conditions elicited greater N400 responses. Moreover, phase synchronization of neural oscillations increased more in incongruent conditions around 400 ms, suggesting that incongruent conditions required sustained effort for cross-modal binding. They also found amplitude increases in the gamma band in centro-parietal within 300 ms of word onset for congruent conditions, suggesting that 11-month-olds process sound-symbolism as perceptual binding. Bien et al.’s (2012) study on cross-modal binding appears to be consistent with Asano et al.’s (2015) findings. Bien et al. (2012) investigated pitch-size mappings rather than linguistic sound-size mappings, and found that cross-modally congruent conditions (i.e., high pitch and small size) elicited a greater P2 response at around 250 ms in intra-parietal regions. Bien et al. (2012) argue that this effect is synaesthetic, and that cross-modal mappings underlie and influence multisensory perception. Taken together, the Bien et al. (2012) and Asano et al. (2015) studies suggest that cross-modally congruent conditions show an early effect in centro-parietal areas which is related to sensory processing, while incongruent conditions show a later effect which is related to semantic integration difficulty. The Kovic et al. (2010) study found a still earlier effect for congruent conditions, but at occipital regions, and moreover was a negative-going rather than positive-going wave. This may be related to the fact that Kovic et al. (2010) trained their participants to learn the mappings first, rather than requiring spontaneous decisions.

The P2 may well be affected by sound-symbolism. In general, the P2 response has been linked to multisensory integration (Bien et al., 2012). In single modality experiments, the auditory P2 response has been linked to higher level categorization processes (Crowley and Colrain, 2004), and auditory processing of linguistic (Carpenter and Shahin, 2013) and non-linguistic information (Shahin et al., 2005). The visual P2 elicited in word reading studies has been linked to phonological and semantic analysis (Landi and Perfetti, 2007; Dien, 2009; Kong et al., 2010). However, the P2 remains relatively poorly understood and is likely to represent several underlying component generation processes, so any functional significance claims regarding the P2 should be taken with caution. Moreover, the topographic distribution of the P2 is not well-established; some studies find a visual P2 elicited in parieto-occipital areas (Freunberger et al., 2007), while others find a more frontal distribution (Key et al., 2005; Barber et al., 2011). As sound-symbolism is related to the expression of other sensory information and may well recruit these senses during language processing, event-related potential (ERP) investigations into multisensory processing are also important to consider. Many of these have centered around visual object naming, recognition, or categorization with respect to congruent and incongruent auditory stimuli (Giard and Peronnet, 1999; Molholm et al., 2002, 2004; Widmann et al., 2004, 2007; Yuval-Greenberg and Deouell, 2007; Bien et al., 2012), but others have hinted at its role in synaesthetic experiences (Brang et al., 2010). Neuroimaging approaches toward ideophones provide evidence for a strong synaesthetic effect between ideophone meaning and brain activation in relevant, non-linguistic areas, while showing that non-words do not elicit similar activity (Osaka et al., 2003, 2004; Osaka and Osaka, 2005, 2009; Osaka, 2009, 2011). These studies argue that the vividness of the mental imagery conjured up by ideophones is due to this ideomotor response across the visual and premotor cortices. These findings suggest a neurological basis for the early definitions (such as Doke, 1935) of ideophones as “vivid.” Kanero et al. (2014) used locomotion and shape ideophones in contrast to arbitrary adverbs and verbs. They found that ideophones activated the right posterior STS, and argue that this area may be the primary location for processing sound-symbolism. They argue that this may also reflect how sound symbolic words function as both linguistic and non-linguistic iconic symbols, while also providing support for hypotheses that sound-symbolism involves cross-domain mapping (Davis, 1961; Martino and Marks, 2001; Ramachandran and Hubbard, 2001).

This study uses naturally occurring ideophones rather than deliberately constructed non-words to investigate sound-symbolism, in particular the suggestion that it is facilitated by synaesthesia-like cross modal mappings. In this study, we recorded ERPs to investigate the temporal characteristics of processing Japanese ideophones in visually presented normal and nonsense sentences. Based on the literature reviewed above, we focused on two ERP responses, the P2 and the N400. We predicted that if significant differences at the P2 between ideophones and arbitrary adverbs were found, it would suggest that phonological processing and/or integration of sensory information in different domains may be involved in the processing of ideophones. If an N400 effect were found, it would suggest that the semantic properties of ideophones are different from those of arbitrary words. Finally, we ran a pilot study run using ten Japanese participants, which showed a P2 response and a sustained late positive response starting at around 400 ms. Accordingly, the focus of the current study was on the P2, N400, and late positive complex responses.

## Materials and Methods

We first carried out a pilot EEG study on a small sample of 10 native Japanese speakers. The task was identical to the main experiment, and was done in order to check the stimuli and to establish whether there was any indication of an effect. Initial analyses of these ten participants confirmed that the P2 and late positive complex were areas of interest for the full experiment. Based on their feedback and corrections, the following stimuli were used for the current experiment.

### Stimuli

Adverbial ideophones with a CVCV–CVCV pattern were the most suitable due to their frequency, variety of meanings, and iconicity of form. Using the Sketchengine program to access the JpWaC online corpus (Kilgarriff et al., 2004), a list of the most frequently used adverbial ideophones was created, and 35 of these were selected for use in the stimuli sentences, as recommended by Kutas et al. (2010). Full stimuli sentences of 3–5 words long were created for these 35 ideophonic adverbs (henceforth referred to as iconic adverbs), as shown in (1) below:

(1) Hanako-ha samusa-de gatagata furueta Hanako-SUBJ cold-with shiver-ICONIC.ADV shake-PAST ‘Hanako shook with shivers because of the cold.’

Thirty-five equivalent arbitrary adverbs were then matched to the iconic adverbs as closely as possible for meaning, total word length, and frequency. Frequency ratings were obtained from the JpWaC corpus; average frequency for ideophones was 2791.9 occurrences in the corpus, average frequency for arbitrary adverbs was 3254.7 occurrences in the corpus, and this was not significantly different (*t*-test, *t* = -1.0886, *p* = 0.284). The first character frequency in each word was also consistent across conditions (*t*-test, *t* = 0.736, *p* = 0.467), and the variety of pitch accents across the stimuli reduces any potential systematic confounds. The same sentences were used for both adverbs, resulting in two conditions – iconic and arbitrary. The iconic and arbitrary adverbs were always located after the first and before the last word in the sentence.

Two further conditions were added to create a behavioral task which would distract participants from the purpose of the experiment. Another verb from a different sentence in the stimulus set was selected in order to make a nonsense sentence in a typical N400 elicitation experiment. Participants were asked to make decisions about whether the sentence meaning was normal or strange. This resulted in 140 sentences spread across four conditions – iconic/sensible, arbitrary/sensible, iconic/nonsense, and arbitrary/nonsense, shown in **Table 1** below.

**Table 1:** Experimental conditions for one set of four example stimuli sentences.

Condition		Example sentence
Iconic	Sensible	Hanako-ha samusa-de gatagata furueta Hanako-SUBJ cold-with shiver-ICONIC.ADV shake-PAST ‘Hanako shook with shivers because of the cold.’
Arbitrary	Sensible	Hanako-ha samusa-de sukoburu furueta Hanako-SUBJ cold-with greatly shake-PAST ‘Hanako shook greatly because of the cold.’
Iconic	Nonsense	Hanako-ha samusa-de gatagata oshieta Hanako-SUBJ cold-with shiver-ICONIC.ADV teach-PAST ?‘Hanako shake-taught because of the cold.’
Arbitrary	Nonsense	Hanako-ha samusa-de sukoburu oshieta Hanako-SUBJ cold-with greatly teach-PAST ?‘Hanako taught greatly because of the cold.’

The 2 × 2 experimental design was chosen for two reasons. Firstly, it enabled a design where the only difference between the sentences was whether or not the adverb was iconic. Secondly, it allowed us to give the participants a sensibility judgment task to keep them focused throughout the experiment. Participants were asked to judge whether the sentences were sensible or nonsense, and to respond by pressing a button accordingly.

It is important to note here that it is the verb which determines whether a sentence is a sensible or nonsense sentence. Therefore, up until the presentation of the sentence-final verb, all sentences are sensible. This means that all ideophones are presented at a point during the sentence where it makes sense, and so the only condition contrast of interest when analyzing the effects of sound-symbolism is whether the word is iconic or arbitrary.

A further 140 filler sentences with no adverbs were also included, giving 280 trials in total. The filler sentences were divided between intransitive, transitive, and ditransitive sentences, and were also split into sensible and nonsense conditions. This further served to disguise the underlying purpose of the experiment. These filler sentences were mostly sourced from dictionary examples.

### Participants

The experiment was carried out on 22 adult Japanese native speaking participants (18 f, 4 m) aged 19–31 with normal or corrected to normal vision. 20 were right-handed, two were left-handed. Participants were recruited from various London universities and were reimbursed with £15 for their time. All participants were educated within the Japanese school system, and are therefore highly proficient readers of Japanese. Ethics approval was obtained from the UCL Research Department of Linguistics Ethics Committee (project ref LING-2013-06-25).

### Procedure

The experiment was conducted in a sound-attenuated, dimly lit room. Participants sat in a chair facing a 17-inch screen situated ~90 cm away. Before each session, participants were given a short practice block. The sentences in the experimental block were randomized and divided into seven blocks of 40 sentences. The order of these blocks was randomized for each participant. Each block lasted ~4 min, and participants were given a short break between each block.

The sentences were presented visually word by word in the center of the screen. Each sentence was preceded by a fixation cross, whose duration was randomized to be between 1900 and 2100 ms. The jitter was included in order to reduce contingent negative variation, a low frequency negative wave elicited when participants expect a stimulus (Luck, 2005). Each word was presented for 1000 ms, and a blank screen was presented between each word for 100 ms. Participants were asked to read the whole sentence, and then decide by a button press whether the sentence was sensible or nonsense after the last word was presented. There were 140 sentences which required “sensible” responses and 140 which required “nonsense” responses. Participants were asked to blink and move between trials but to stay as still as possible during trials.

Words were generally presented in the most natural Japanese script; that is, Jōyō kanji were used for verbs, adjectives, and nouns, katakana for loanwords, and hiragana for adverbs, ideophones, and grammatical functions. Written sentences were double checked with a native speaker, and then refined with 10 more native speakers in the pilot test. There were some disagreements between native speakers over the naturalness of some sentences, but all sentences were considered acceptable before the presentation of the final verb. We therefore do not expect to find a main effect of sentence sense in ERPs taken at the presentation of the ideophone or arbitrary adverb.

### EEG Recording and Analysis

The continuous EEG was recorded using 64 electrodes fixed to an elastic cap (Biosemi; http://www.biosemi.com/headcap.htm) using the 10–10 system. All electrode offsets were kept within ±20 mV as recommended by Biosemi. All EEG and external channels were amplified using a Biosemi ActiveTwo DC amplifier. EEG-waveforms were time-locked to the onset of the presentation of the orthographic stimulus.

The electro-oculogram was recorded from two external electrodes; one below the left eye to measure blinks and vertical eye movements (VEOG), and one at the right canthus to measure horizontal eye movements (HEOG). All electrodes were referenced off-line to the average of left and right mastoids.

EEG data were filtered with 0.5–30 Hz bandpass oﬄine. An independent component analysis (ICA) implemented in EEGLAB v. 12.0.2.04b (Delorme and Makeig, 2004) was run on all participants’ data and horizontal and vertical eye-movements as well as blinks were removed from the data. Trials which still contained artifacts were rejected oﬄine using the ERPLAB v. 3.0.2.1 (Lopez-Calderon and Luck, 2014) artifact detection tools. The moving window peak-to-peak threshold tool (moving window width: 200 ms, voltage threshold: 100 μV, window step: 20 ms) and the step-like artifacts tool (moving window width: 400 ms, voltage threshold: 35 μV, window step: 10 ms) were used to reject trials with these artifacts.

One participant was excluded from the analysis due to heavy sweating during the experiment making the data unusable. In the remaining 21 participants, 18.9% of critical trials were excluded. In three participants, readings for bad electrodes (F6; PO4 and PO8; POz, PO4, and PO8 in the three participants respectively) were not included in the ICA and were interpolated from adjacent electrodes before artifact detection.

Averaged ERPs were calculated per condition per participant from 200 ms pre-onset to 800 ms post-onset of each stimulus word. These were then grouped into critical bins for the four experimental conditions; iconic adverbs in sensible sentences, iconic adverbs in nonsense sentences, arbitrary adverbs in sensible sentences, and arbitrary adverbs in nonsense sentences.

The P2 response was calculated with an automatic peak-detection procedure in the 200–300 ms window from the grand average, and the resulting mean amplitudes within a 2 ms interval around each peak were used for further statistical analysis. The identified peak was 254 ms, and so a window of 252–256 ms was used for analyses. This procedure was used in order to match the analyses in Bien et al. (2012) as closely as possible; the similar latencies of the resulting peaks (254 and 250 ms) suggest that the same response has been identified. Repeated measures 2 × 2 × 64 ANOVAs were computed to investigate three factors; iconicity, sense, and electrode location. The factor of iconicity had two levels, iconic and arbitrary. The factor of sense also had two levels, sensible and nonsense. The factor of electrode location had 64 levels, which were all electrode locations from which EEG recordings were taken. In order to get a broader picture of the distribution of the effect across the scalp, we also performed a quadrant analysis. After excluding midline electrodes, we grouped the remaining 44 electrodes into four quadrants of 11: left anterior (Fp1, AF7, AF3, F7, F5, F3, F1, FT7, FC5, FC3, FC1), right anterior (Fp2, AF4, AF8, F2, F4, F6, F8, FC2, FC4, FC6, FT8), left posterior (TP7, CP5, CP3, CP1, P7, P5, P3, P1, PO7, PO3, O1), and right posterior (CP2, CP4, CP6, TP8, P2, P4, P6, P8, PO4, PO8, O2). A repeated measures 2 × 2 × 4 ANOVA was then computed.

The N400 response was calculated more generally, taking the mean amplitude across a time window of 350–550 ms. This slightly later time window was taken in order to remain consistent with existing Japanese sound-symbolic ERP literature (Asano et al., 2015). The same repeated measures 2 × 2 × 64 ANOVAs were computed to investigate three factors; iconicity, sense, and electrode location. As the data did not resemble a typical N400 response but rather a long-lasting increased positivity which appeared to begin at around 400 ms and last until the end of the trial, we also tested the time window of 400–800 ms in the same way.

## Results

Behavioral results showed that participants made errors on 7.09% of trials. The individual error rate ranged from 2.5 to 16.8%, so no participant was excluded on the basis of high error rate. Mistakes were broadly consistent across the main conditions, with participants making mistakes on 7.41% of iconic sentences and 11.77% of arbitrary sentences. This was not significantly different (*t* = 1.61, *p* = 0.11). Eight sentences were miscategorized by over half the participants. One sentence was a filler sentence taken from the dictionary (Shogakukan, 2001), and seven were critical trials. However, as behavioral responses were unrelated to the underlying purpose of the experiment, and as it was the sentence-final verb which dictated the sensibility of a sentence whereas the ERP analysis was conducted on target adverbs presented before the final verbs, incorrect responses were not excluded from the iconic/arbitrary target word analysis.

**FIGURE 1:**
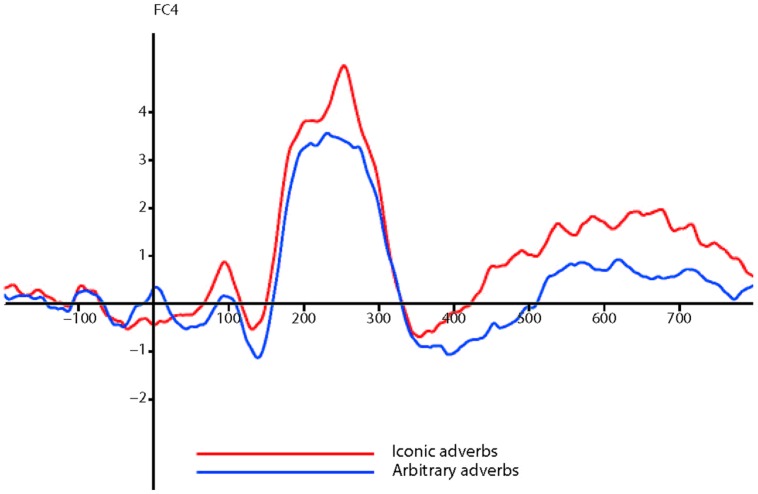
**Event-related potential (ERPs) in response to iconic and arbitrary adverbs**.

In the P2 analysis, there was a significant difference between ERP amplitudes elicited by iconic and arbitrary conditions (*F* = 10.095, df = 1,20, *p* = 0.005, $\eta_{p}^{2}$ = 0.335), as shown in **Figure 1**.

The interaction between iconicity and electrode location only tended toward significance (*F* = 2.045, *p* = 0.088). In order to get a broader picture of the distribution of the effect across the scalp, we also performed a quadrant analysis. After excluding midline electrodes, we grouped the remaining 44 electrodes into four quadrants of 11; left anterior, right anterior, left posterior, and right posterior.

A repeated measures 2 × 2 × 4 ANOVA (iconicity, sense, and quadrant) across the same 252–256 ms window showed that the significant factors were iconicity (*F* = 11.357, df = 1,20, *p* = 0.003, $\eta_{p}^{2}$ = 0.362) and quadrant (*F* = 34.287, df = 1,20, *p* < 0.001, $\eta_{p}^{2}$ = 0.632). However, there was no significant interaction between iconicity and quadrant, which suggests that no generalizations can be made about the scalp topography of the effect of iconicity from the ANOVA. The actual difference in ERPs between conditions was ~1 μV at frontal sites. Waveforms and topographic plots suggested that the effect was stronger in the anterior quadrants. As predicted, there was no significant main effect of sense.

**FIGURE 2:**
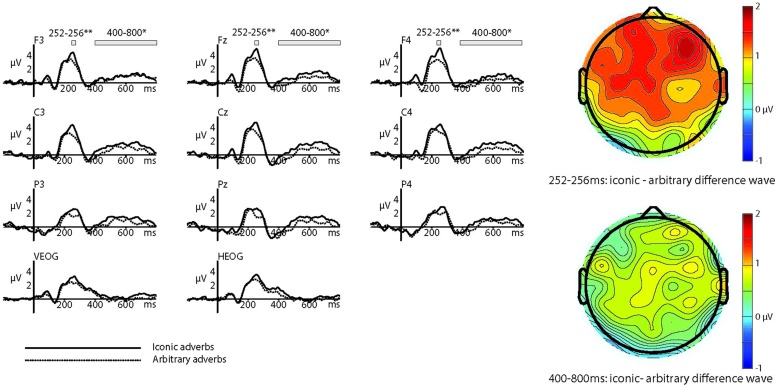
**Event-related potentials and topoplots in response to iconic and arbitrary adverbs**.

**FIGURE 3:**
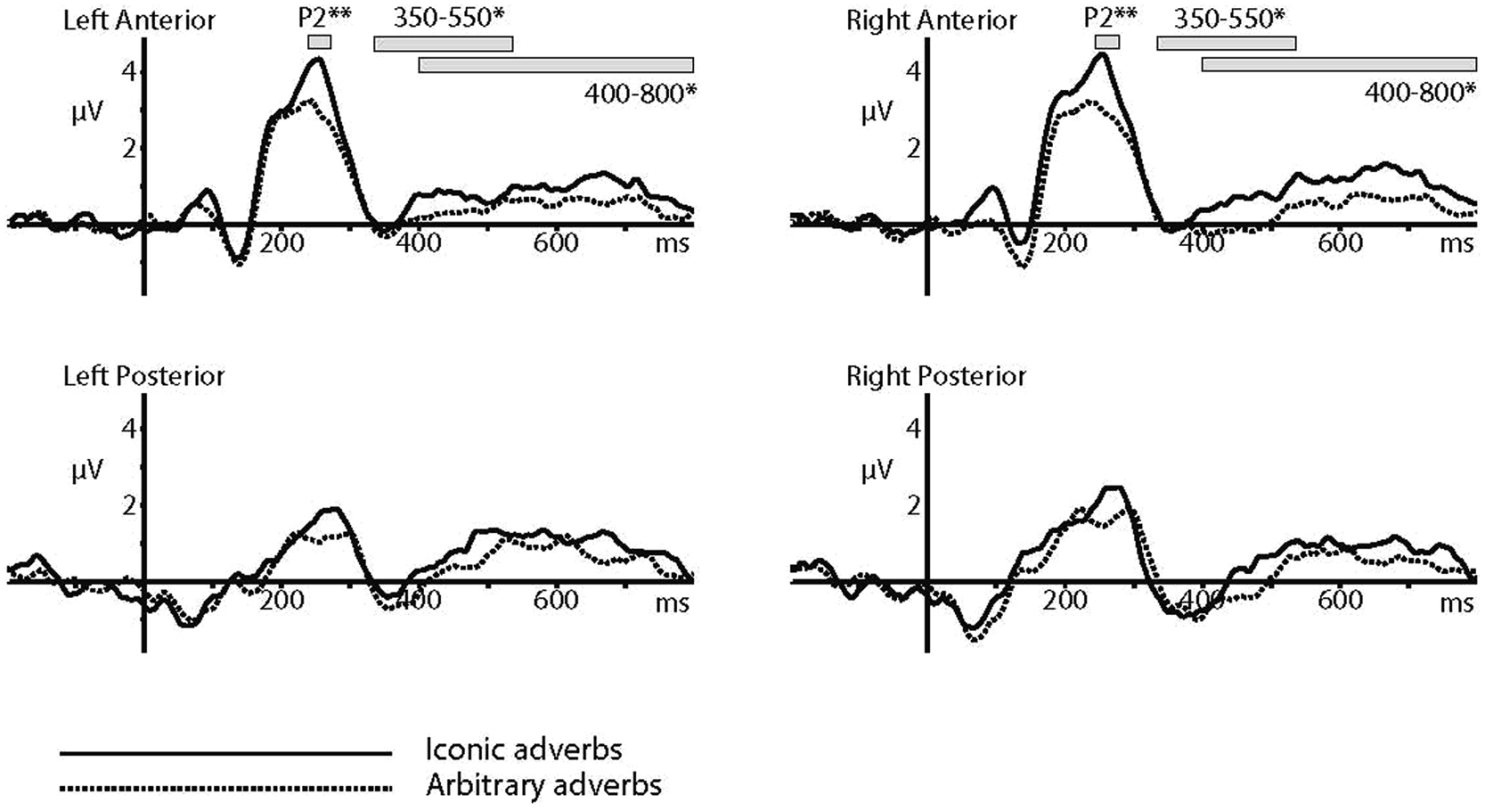
**Event-related potentials in response to iconic and arbitrary adverbs – 4 quadrants**.

In the N400 analysis, there was a significant difference between ERP amplitudes elicited by iconic and arbitrary conditions across the time window of 350–550 ms (*F* = 7.566, df = 1,20, *p* = 0.012, $\eta_{p}^{2}$ = 0.274). Again, there were no other significant main effects or significant interactions. As the long-lasting increased positivity appeared to begin at around 400 ms and last until the end of the trial, we also tested the time window of 400–800 ms. This too was significant (*F* = 5.351, df = 1,20, *p* = 0.031, $\eta_{p}^{2}$ = 0.211), and there were no significant interactions. The two main effects can be seen in the ERPs and topoplots in **Figure 2** and in the ERPs across the four quadrants shown in **Figure 3** below.

Finally, if the effect was simply because of the reduplication, in that there was a visual and/or orthographic difference between the ideophones and the arbitrary words, then non-Japanese reading participants should also be sensitive to the visual reduplication. We conducted a control experiment with 34 native Dutch speakers aged 19–27 (24 f, 10 m) to establish whether the effect was due to the reduplicated nature of the ideophones. Participants saw the adverbs across the two conditions while performing an N-back task. There was no effect either at the P2 (*F* = 0.28, *p* = 0.60) or late positive complex (*F* = 0.92, *p* = 0.34) windows.

## Discussion

The present study provides additional electrophysiological evidence that sound-symbolism in natural language modulates the P2 response, which in this study is the visual P2. Participants were presented with short Japanese sentences one word at a time. They were instructed to make sensibility judgments on the stimuli, unaware that the experiment was in fact designed to measure their responses to sound-symbolic ideophones and arbitrary adverbs. The ERP data showed that participants’ sensitivity to the ideophone manipulation was not affected by the sensibility judgment task, and revealed different brain signatures elicited by sound-symbolic and non-sound-symbolic conditions in two windows; the P2 with a peak at 254 ms, and the late effect between 400 and 800 ms. This is in line with some previous ERP studies on cross-modal congruency (Bien et al., 2012; Asano et al., 2015), but not others (Kovic et al., 2010). Since the experimental literature on sound-symbolism is still relatively sparse and reports a variety of effects, our findings are an interesting addition to the existing literature, but any theoretical claims we make based upon them can only be exploratory. We have found that ideophones elicit a greater P2 response than arbitrary adverbs, and that there is a long-lasting late effect which we take to be a late positive complex in response to the ideophones (rather than a late negative complex or elongated N400 in response to the arbitrary adverbs). We theorize that the distinctive phonology of ideophones triggers a sensory integration process of sound and the sensory information which the ideophone activates by association, which elicits the larger P2 effect, while the late positive complex may reflect the more effortful retrieval of ideophones in comparison to arbitrary words. However, word imageability and concreteness effects may also be part of this process.

The visual P2 is related to auditory input, phonological processing, and multisensory integration (Dien, 2009; Bien et al., 2012). Both of these are important factors when it comes to analyzing the processing of ideophones, as ideophones have distinctive phonological structures (Hamano, 1998; Bodomo, 2006; Dingemanse, 2012) and typically depict sensory imagery in a quasi-synaesthetic way (Kita, 1997; Hinton et al., 2006; Dingemanse, 2012), in that speakers are aware that the form of the word naturally matches the word’s meaning. In this experiment, it is plausible that the combination of phonological processing and a sensory integration process between sound and the triggered sensory information contributes to the heightened P2 here. The ideophones’ phonological salience alone is unlikely to be the sole driver of the effect. The most obvious difference between the ideophones and the arbitrary adverbs used in this experiment was the reduplicated structure, but this also appears (albeit non-productively) in common arbitrary adverbs. It is similarly unlikely that the main factor is the individual segmental sound-symbolic properties of ideophones (Hamano, 1998), as this cannot explain the absence of the same effect for arbitrary words which use the same segments. Moreover, a control experiment with non-Japanese speaking Dutch participants ruled out any strictly visual and/or orthographic contribution to the effect. Rather, we argue that the reduplication and the specific position of certain segments, as well as the special prosodic foregrounding often assigned to ideophones (Dingemanse, 2013), results in a more holistic distinctive phonology for ideophones. This allows the speaker to recognize that this is an ideophone, a special word which depicts sensory imagery and therefore needs to be processed slightly differently. This activates a second sensory system by association, and leads to an integration process of the sound of the ideophone and the sensory information conveyed by the ideophone, in a whole process which may be considered (quasi-) synaesthetic. The ideophone-synaesthetic effect is consistent with the linguistic literature on the vivid experience of ideophones, as well as the various studies by the Osaka group, where ideophones were shown to activate the sensory areas of the brain which corresponded to the ideophones’ meaning. It also echoes Brang et al.’s (2010) point that the P2 reflects synaesthetic experience. However, we prefer to remain agnostic over whether, and if so how far, cross-modal correspondences in language fall into the spectrum of synaesthesia.

One limitation of this study is that words were presented visually, while previous literature used auditory or concurrent audio/visual stimuli. As reading automatically activates phonological representations even in skilled readers (Ziegler and Goswami, 2005; Savill et al., 2011), it is not implausible to discuss phonological processing from an experiment which presented orthographic stimuli, although it is not possible to make direct comparisons with Asano et al.’s (2015) aurally presented non-words. Rather than claiming the same response reflecting the same processes, we claim to identify a similar response reflecting similar processes. Likewise, directly comparing linguistic stimuli in this study with non-linguistic stimuli in the Bien et al. (2012) study would be disingenuous. However, we argue that similar underlying processes are at play. Bien et al. (2012) found that congruent cross-modal mappings elicited a larger P2 response, which was diminished after using TMS on the right intraparietal area hypothesized to be responsible for multisensory integration. Bien et al. (2012) link an increased P2 response with congruent multisensory integration between two sensory modalities; we follow that interpretation in suggesting that our results represent the sensory integration between sound and coactivated sensory information.

The present study also appears to contrast with audio-visual multisensory integration processes where hearing speech and viewing congruent speech articulation results in a decreased P2 amplitude when compared to unimodal presentation of hearing speech alone (Wassenhove et al., 2005). However, the two modalities used in typical audio-visual integration studies involve two sensory aspects of one external audio-visual object, and the two modalities directly represent the same object. On the other hand, this study and Bien et al.’s (2012) study compare two sensory aspects from two separate origins; sound and size in Bien et al. (2012), and sound and elicited-by-association sensory information in this study. It is not implausible that multisensory integration processes are different depending on whether the senses being integrated are coming from the same source or not. A further limitation of this study was that we were not able to obtain imageability and concreteness ratings for all the stimuli used in both conditions, as adverbs in general and ideophones particularly are not used in rating studies as often as nouns and verbs. Given that descriptions of ideophones in the literature refer to their vividness and intensity, it could well be that word imageability may contribute to the effects found here. Similarly, orthographic neighborhood was not calculated and could be a confound, but this seems unlikely as orthographic neighborhood tends to be linked to the N400 component rather than the P2 or late positive complex (Holcomb et al., 2002). Finally, another potential interpretation is that the P2 effect represents a visual process. The visual P2 has also been linked to various cognitive processes involving memory in priming tasks (Freunberger et al., 2007), as well as visual features in selective attention tasks with higher amplitudes elicited by expected visual features (Federmeier and Kutas, 2002). However, this experiment was designed with no additional working memory task or visual feature detection task, the memory requirements were limited and the visual features of the adverbs were consistent across trials, and a control experiment with non-Japanese speaking Dutch participants failed to find any visual effect. Therefore, a phonological processing and sensory integration interpretation of the P2 effect here appears most likely.

One final issue to discuss concerning the P2 in this study is its scalp topography. Given the various scalp topographies found in the general P2 literature and in the cross-modal mapping literature, we made no specific predictions. We found that the effect in our study was not restricted to any particular electrodes or quadrants, but appeared to be stronger at frontal and fronto-central areas. This is somewhat inconsistent with the P2 findings of Bien et al. (2012) and the 1–300 ms gamma band increase in Asano et al. (2015), who localized their effects to centro-parietal regions. We argue that this is related to how we presented the stimuli; as the visual P2 tends to be maximally frontal while the auditory P2 tends to be maximally central (Key et al., 2005), it is perhaps not surprising that this study, which presented visual words only, found an apparently more frontal effect compared to studies which presented auditory and visual stimuli. None of these three studies is consistent with Kovic et al. (2010), who found an occipital effect of sound-symbolism. Moreover, their effect was ~100 ms earlier and was a negative-going wave. We can only speculate as to the reasons for the threefold difference between Kovic et al. (2010) and the present study, Bien et al. (2012) and Asano et al. (2015), but this difference may arise from the fact that participants in Kovic et al. (2010) had been trained to memorize congruent and incongruent mappings, whereas the other studies relied on spontaneous judgments based on intuition or native language.

The N400 test in the 350–550 ms timeframe was significant, which would at first glance echo Asano et al.’s (2015) finding that sound-symbolism facilitates semantic integration. However, with the possible exception of the waveforms in the left posterior quadrant, the response in this study does not resemble an N400 response, and so to discuss our significant findings as if they were typical N400s would be disingenuous. Rather, our results more closely resemble a late sustained positive complex, and in this section we speculate as to what this may mean. The late sustained positive complex in response to ideophones may reflect the necessity for more post-lexical processing (Lau et al., 2008), due to an increased processing cost of sound-symbolism in language. Ideophones in Japanese have high frame specificity and can have non-standard syntactic requirements (Akita, 2012; Dingemanse, 2012), meaning that the syntactic integration of ideophones into a sentence is perhaps harder than it is for arbitrary words. This late positive complex may be a neural representation of the trade-off between the expressivity of sound-symbolism and the efficiency of arbitrariness.

One further issue which future studies should address is the precise mechanism of the putative synaesthetic effect; why should phonologically distinct ideophones (and not, say, the phonological distinctiveness of loan words which use foreign phonology) trigger a sensory integration effect? It appears that various factors contribute to the sound-symbolism of ideophones; consonants, vowels, syllabic structure, and prosody, as well as the semantic domain of sensory imagery that they express. The combination of some or all of these factors is probably what makes ideophones special, while having just one factor is not enough to make other words sufficiently sound-symbolic. It remains to be seen whether Japanese speakers’ sensitivity to these special factors is simply statistically learned (and therefore making Japanese ideophones examples of conventional sound-symbolism) or whether there is some degree of inherent sound-symbolism to these factors; the behavioral experiments on English speakers with no knowledge of Japanese statistical patterns would suggest that it is the latter (Oda, 2000; Iwasaki et al., 2007a,b; Kantartzis et al., 2011; Yoshida, 2012), although there is always some inevitable conventionalism of ideophones’ form within any given language. The replication of this study using ideophones in a different language would go some way toward answering this.

## Conclusion

This study has shown that sound-symbolic adverbs in Japanese have a significant effect on the P2 response and in the 400–800 ms timeframe when compared with arbitrary adverbs, and we speculate that this effect may be due to the distinctive phonological properties of ideophones precipitating a sensory integration process between sound of the ideophones and the sensory representations of the triggered sensory domains. Further research is needed to clarify whether this effect is generalizable to all ideophones, and whether this effect can be replicated with speakers of other languages. However, this study provides exciting evidence that sound-symbolism is not just psycholinguistically detectable in deliberately constructed non-words, but also in real sound-symbolism in natural language.

## Conflict of Interest Statement

The Editor Gabriella Vigliocco declares that, despite being affiliated to the same institution as the authors Gwilym Lockwood and Jyrki Tuomainen, the review process was handled objectively. The authors declare that the research was conducted in the absence of any commercial or financial relationships that could be construed as a potential conflict of interest.
