# Supplementary material for: Ideophones in Japanese modulate the P2 and late positive complex responses
Source: Front Psychol. 2015 Jul 2;6:933. doi: 10.3389/fpsyg.2015.00933 (PMC4488605; doi:10.3389/fpsyg.2015.00933)
Supplement: Supplementary file 1 [file Data_Sheet_1.DOCX]

**Appendix 1: iconic/arbitrary sentences**

Iconic? Congruent? 日本語 English

Y Y 花火がどんどんと上がった The fireworks went up with a bang.

N Y 花火が同時に上がった The fireworks went up at the same time.

Y N 花火がどんどんと食べた The fireworks ate with a bang.

N N 花火が同時に食べた The fireworks ate at the same time.

Y Y ひろはそこそこに勉強をし始めた Hiro immediately started studying.

N Y ひろは直ちに勉強をし始めた Hiro immediately started studying.

Y N ひろはそこそこに死に始めた Hiro immediately started dying.

N N ひろは直ちに死に始めた Hiro immediately started dying.

Y Y 花子はついつい秘密を教えた Hanako told a secret without meaning to.

N Y 花子は思わず秘密を教えた Hanako told a secret without thinking.

Y N 花子はついつい秘密を飲んだ Hanako drank a secret without meaning to.

N N 花子は思わず秘密を飲んだ Hanako drank a secret without thinking.

Y Y 花子はどきどきして手紙を開けた Hanako opened the letter with her heart beating.

N Y 花所は感動して手紙を開けた Hanako opened the letter in excitement.

Y N 花子はどきどきして手紙を運転した Hanako drove the letter with her heart beating.

N N 花子は感動して手紙を運転した Hanako drove the letter in excitement.

Y Y 子供は庭でわくわくと走った The children excitedly ran around in the garden.

N Y 子供は庭でうれしく走った The children happily ran around in the garden.

Y N 子供は庭でわくわくと寝た The children excitedly slept in the garden.

N N 子供は庭でうれしく寝た The children happily slept in the garden.

Y Y ひろはいらいらと部屋を飛び出した Hiro angrily flew out of the room.

N Y ひろは怒って部屋を飛び出した Hiro angrily flew out of the room.

Y N ひろはいらいらと部屋を教えた Hiro angrily informed the room.

N N ひろは怒って部屋を教えた Hiro angrily informed the room.

Y Y 星が空にきらきらと輝く The stars shine brightly in the sky.

N Y 星が空に絶えず輝く The stars shine constantly in the sky.

Y N 星が空にきらきらと泳ぐ The stars swim brightly in the sky.

N N 星が空に絶えず泳ぐ The stars swim constantly in the sky.

Y Y ひろは電車にぎりぎり間に合った Hiro just barely made the train.

N Y ひろは電車にかろうじて間に合った Hiro just barely made the train.

Y N ひろは電車にぎりぎり曲がった Hiro just barely turned to the train.

N N ひろは電車にかろうじて間に合った Hiro just barely turned to the train.

Y Y 花子は手紙をばらばらに引き裂いた Hanako tore the letter up into pieces.

N Y 花子は手紙を一気に引き裂いた Hanako tore the letter up at once.

Y N 花子は手紙をばらばらに噛んだ Hanako bit the letter into pieces.

N N 花子は手紙を一気に噛んだ Hanako bit the letter at once.

Y Y ひろはいやいや先生の命令に従った Hiro reluctantly followed his teacher's orders.

N Y ひろは無理やりに先生の命令に従った Hiro followed his teacher's orders against his will.

Y N ひろはいやいや先生の命令に間に合った Hiro reluctantly was in time for his teacher's orders.

N N ひろは無理やりに先生の命令に間に合った Hiro was in time for his teacher's orders against his will.

Y Y ひろは町の様子をいきいきと描いた Hiro painted the town's scene vividly.

N Y ひろは町の様子をせつじつに描いた Hiro painted the town's scene accurately.

Y N ひろは町の様子をいきいきと飛び出した Hiro flew out of the town's scene vividly.

N N ひろは町の様子をせつじつに飛び出した Hiro flew out of the town's scene accurately.

Y Y ひろは恋人についておいおい泣いた Hiro cried bitterly over his girlfriend.

N Y ひろは恋人について大変泣いた Hiro cried greatly over his girlfriend.

Y N ひろは恋人についておいおい踊った Hiro danced bitterly about his girlfriend.

N N ひろは恋人について大変踊った Hiro danced greatly about his girlfriend.

Y Y 花子は図書館でがんがん勉強した Hanako studied like mad in the library.

N Y 花子は図書館で大変勉強した Hanako studied a lot in the library.

Y N 花子は図書館でがんがん育った Hanako grew up like mad in the library.

N N 花子は図書館で大変育った Hanako grew up a lot in the library.

Y Y 花子が腕をぐるぐる回した Hanako waved her arm round and round

N Y 花子が腕をすぐに回した Hanako waved her arm suddenly.

Y N 花子が腕をぐるぐる聞いた Hanako listened to her arm round and round

N N 花子が腕をすぐに聞いた Hanako listened to her arm suddenly.

Y Y 花子は仕事でばりばり働く Hanako works hard at her job.

N Y 花子は仕事でせつじつに働く Hanako works properly at her job.

Y N 花子は仕事でばりばりサボる Hanako skives off hard at her job.

N N 花子は仕事でせつじつにサボる Hanako skives off properly at her job.

Y Y 床に豆がぼろぼろ散らばっていた Beans were spilt on the floor, scattered everywhere.

N Y 床に豆がそこら中散らばっていた Beans were spilt all over the floor.

Y N 床に豆がぼろぼろ書いていた Beans were drawn on the floor, scattered everywhere.

N N 床に豆がそこら中書いていた Beans were drawn all over the floor.

Y Y ひろはだらだらと仕事をする Hiro does his job inefficiently.

N Y ひろはきらくに仕事をする Hiro does his job carelessly.

Y N ひろはだらだらと仕事を見る Hiro looks at his job inefficiently.

N N ひろはきらくに仕事を見る Hiro looks at his job carelessly.

Y Y ひろがにこにこと冗談を言った Hiro told the story with a grin.

N Y ひろがうれしく冗談を言った Hiro told the story happily.

Y N ひろがにこにこと冗談を登った Hiro climbed up the story with a grin.

N N ひろがうれしく冗談を登った Hiro climbed up the story happily.

Y Y のぼりが風でばたばたはためいている The flag flaps around in the wind.

N Y のぼりが風でしきりにはためいている The flag flaps repeatedly in the wind.

Y N のぼりが風でばたばた寝る The flag sleeps around in the wind.

N N のぼりが風でしきりに寝る The flag sleeps repeatedly in the wind.

Y Y ひろはこつこつと勉強した Hiro studied determinedly.

N Y ひろはしきりに勉強した Hiro studied constantly.

Y N ひろはこつこつと死んだ Hiro died determinedly.

N N ひろはしきりに死んだ Hiro died constantly.

Y Y ひろはぶらぶらと歩いた Hiro walked slowly/"strolling-ly".

N Y ひろはきらくに歩いた Hiro walked casually.

Y N ひろはぶらぶらと開けた Hiro opened slowly/"strolling-ly".

N N ひろはきらくに開けた Hiro opened casually.

Y Y ひろはふらふらと立ち上がった Hiro stood up unsteadily.

N Y ひろはぎこちなく立ち上がった Hiro stood up clumsily.

Y N ひろはふらふらと育った Hiro grew up unsteadily.

N N ひろはぎこちなく育った Hiro grew up clumsily.

Y Y 花子は論文をぼちぼち書いた Hanako gradually wrote her thesis.

N Y 花子は論文をしきりに書いた Hanako constantly wrote her thesis.

Y N 花子は論文をぼちぼち食べた Hanako gradually ate her thesis

N N 花子は論文をしきりに食べた Hanako constantly ate her thesis

Y Y スケーターはアイスリンクでくるくる回った The skater turned around in circles on the ice rink

N Y スケーターはアイスリンクでしきりに回った The skater turned around constantly on the ice rink

Y N スケーターはアイスリンクでくるくる食べた The skater ate in circles on the ice rink

N N スケーターはアイスリンクでしきりに食べた The skater ate constantly on the ice rink

Y Y 花子はページをぱらぱらとめくった Hanako turned the pages with a flourish.

N Y 花子はページをちくじにめくった Hanako turned the pages one after the other.

Y N 花子はページをぱらぱらと飲んだ Hanako drank the pages with a flourish.

N N 花子はページをちくじに飲んだ Hanako drank the pages one after the other.

Y Y ひろはごくごくと水を飲んだ Hiro drank the water in gulps.

N Y ひろはせつに水を飲んだ Hiro drank the water eagerly.

Y N ひろはごくごくと水を書いた Hiro wrote the water in gulps.

N N ひろはせつに水を書いた Hiro wrote the water eagerly.

Y Y 花子は涙がはらはらと流れた Hanako's tears poured out in streams.

N Y 花子は涙がやたらに流れた Hanako's tears poured out excessively.

Y N 花子は涙がはらはらと歩いた Hanako's tears walked in streams.

N N 花子は涙がやたらに歩いた Hanako's tears walked excessively.

Y Y 花子が俳句をさらさらと語った Hanako recited a haiku fluently.

N Y 花子が俳句をりゅうちょうに語った Hanako recited a haiku fluently.

Y N 花子が俳句をさらさらと走った Hanako ran a haiku fluently.

N N 花子が俳句をりゅうちょうに走った Hanako ran a haiku fluently.

Y Y ひろはのびのびと育った Hiro grew up free from all cares.

N Y ひろはきらくに育った Hiro grew up comfortably.

Y N ひろはのびのびと心配した Hiro worried free from all cares.

N N ひろはきらくに心配した Hiro worried comfortably.

Y Y ひろはいうことをころころと変える Hiro changes his mind randomly.

N Y ひろはいうことをきまぐれに変える Hiro changes his mind at whim.

Y N ひろはいうことをころころと買う Hiro buys what he says randomly.

N N ひろはいうことをきまぐれに買う Hiro buys what he says at whim.

Y Y 花子は寒さでがたがた震えた Hanako shook with shivers because of the cold.

N Y 花子は寒さですこぶる震えた Hanako shook a lot becaue of the cold.

Y N 花子は寒さでがたがた教えた Hanako taught with shivers because of the cold.

N N 花子は寒さですこぶる教えた Hanako taught a lot because of the cold.

Y Y 休みはずるずると過ぎてしまった The holiday slipped away.

N Y 休みはいつしか過ぎてしまった The holiday passed by before I realised it.

Y N 休みはずるずると働いた The holiday worked in a slippery fashion.

N N 休みはいつしか働いた The holiday worked before I realised it.

Y Y ひろはにんじんをぶつぶつと輪切りにした Hiro cut the carrot into tiny pieces.

N Y ひろはにんじんをみじかく輪切りにした Hiro cut the carrot up small.

Y N ひろはにんじんをぶつぶつと聞いた Hiro listened to the carrot into tiny pieces.

N N ひろはにんじんをみじかく聞いた Hiro listened to the carrot until it was small.

Y Y 子供は外でわいわい騒いだ The children shouted in uproar outside.

N Y 子供は外でうるさく騒いだ The children shouted noisily outside.

Y N 子供は外でわいわい勉強した The children studied in uproar outside.

N N 子供は外でうるさく勉強した The children studied noisily outside.

Y Y 花子はぺらぺらとフランス語を話す Hanako speaks French fluently.

N Y 花子はりゅうちょうにフランス語を話す Hanako speaks French fluently.

Y N 花子はぺらぺらとフランス語を洗う Hanako washes French fluently.

N N 花子はりゅうちょうにフランス語を洗う Hanako washes French fluently.
